# Factors perceived by health professionals to be barriers or facilitators to caries prevention in children: a systematic review

**DOI:** 10.1186/s12903-023-03458-1

**Published:** 2023-10-19

**Authors:** Guillemette Lienhart, Masson Elsa, Pierre Farge, Anne-Marie Schott, Beatrice Thivichon-Prince, Marc Chanelière

**Affiliations:** 1 Service d’Odontologie, Hospices Civils de Lyon, 6/8 Place Deperet, 69007 Lyon, France; 2 Hôpital Femme Mère Enfant, Hospices Civils de Lyon, 59 Boulevard Pinel, 69500 Bron, France; 3 Research On Healthcare Performance (RESHAPE), INSERM U1290, Université Claude Bernard Lyon 1, Domaine Rockefeller, 8 Avenue Rockefeller, 69373 Lyon 8, France

**Keywords:** Dental caries, Children, Health promotion, Systematic review, Attitude of health personnel, Primary care, Barriers, Facilitators, Behavior change

## Abstract

**Background:**

Considered the most prevalent noncommunicable disease in childhood, dental caries is both an individual and a collective burden. While international guidelines highlight prevention as a major strategy for caries management in children, health professionals still struggle to implement prevention into their clinical practice. Further research is needed to understand the gap between the theoretical significance of dental prevention and its lack of implementation in the clinical setting. This systematic review aims to identify and classify factors perceived by health professionals to be barriers or facilitators to caries prevention in children.

**Method:**

A systematic literature search was conducted in three electronic databases (Medline, Web of Science and Cairn). Two researchers independently screened titles, abstracts and texts. To be selected, studies had to focus on barriers or facilitators to caries prevention in children and include health professionals as study participants. Qualitative and quantitative studies were selected. The factors influencing caries prevention in children were sorted into 3 main categories (clinician-related factors, patient-related factors, and organizational-related factors) and then classified according to the 14 domains of the theoretical domains framework (TDF).

**Results:**

A total of 1771 references were found by combining manual and database searches. Among them, 26 studies met the inclusion criteria, of which half were qualitative and half were quantitative studies. Dentists (*n* = 12), pediatricians (*n* = 11), nurses (*n* = 9), and physicians (*n* = 5) were the most frequently interviewed health professionals in our analysis. Barriers and facilitators to caries prevention in children were categorized into 12 TDF domains. The most frequently reported domains were *Environmental Context and Resources*, *Knowledge* and *Professional Role and Identity.*

**Conclusion:**

This systematic review found that a wide range of factors influence caries prevention in children. Our analysis showed that barriers to pediatric oral health promotion affect all stages of the health care system. By highlighting the incompatibility between the health care system’s organization and the implementation of caries prevention, this study aims to help researchers and policy-makers design new interventions to improve children’s access to caries prevention.

**Trial registration:**

PROSPERO CRD42022304545.

**Supplementary Information:**

The online version contains supplementary material available at 10.1186/s12903-023-03458-1.

## Background

Untreated caries in deciduous teeth affected nearly half a billion children worldwide in 2017 [1] and is considered the most prevalent noncommunicable disease in childhood [2]. In addition to the economic burden [3, 4], dental caries and its complications have a negative impact on family activities, children’s and parents’ well-being [5, 6], children’s future oral health [7, 8] and quality of life [9, 10]. Carious lesions result from the demineralization of dental hard tissues by acid production derived from the metabolization of fermentable carbohydrates by specific bacteria found in dental plaque [11]. Dental caries is a chronic multifactorial disease caused by complex interactions of genetic, biochemical, anatomical, social, and behavioral factors. Given that poor brushing leads to the development of dental plaque and frequent sugar intake sustains the metabolism of acidogenic bacteria, patient health behaviors are critical etiologic factors [12, 13]. Thus, the management strategy for dental caries is based on a mixed approach combining the treatment of cavitated and noncavitated lesions with the prevention of recurrence and occurrence of new lesions through the control of risk factors.

Oral health promotion in children involves to consider multiple determinants including the actors, the healthcare system as well as the general environment (social and cultural context, living environment, etc.). In this article, the authors are focusing on the actors and the system organization. At this level, oral health prevention relies on a comprehensive patient-centered approach in which clinical decision-making is based on the assessment of the child’s individual risk factors [14, 15]. Identifying the patient’s specific needs leads to the adoption of local measures, such as fluoride varnish application and fissure sealants, as well as lifestyle measures aimed at encouraging twice-daily brushing and a low sugar diet [16]. Behavioral measures cover a wide range of interventions, from chairside talks to complex educational programs built on chronic disease management or behavior change theories [17, 18]. Currently, all international guidelines [14, 16, 19] highlight prevention as a key strategy for caries management in children. Although fluoride varnish and sealants have long proven to be effective [20, 21], some authors consider sugar the main etiological factor in the carious process [12], with findings suggesting a lower risk of dental caries when free-sugar intake is less than 10% of total energy intake [22].

Because oral health is an integral component of overall health, the provision of dental preventive activities is the role and responsibility of dental professionals (dentists, hygienists, dental nurses, etc.) and other primary care providers involved in the child’s overall care (pediatricians, family physicians, nurses, social workers, midwives, etc.). Several studies report that family physicians and pediatricians strongly recognize the importance of their role in children’s oral health promotion [23, 24]. However, their clinical practice does not appear consistent with this favorable statement. According to various cross-sectional studies, 50 to 75% of physicians would not assess children’s risk for dental caries [25, 26], more than 23% would not provide diet counseling [24, 25], and less than 10% would apply fluoride varnish to high caries-risk children [26–28]. Considering dentists, studies also indicate that their daily practices do not strongly emphasize prevention. Practitioners report spending little time on patient education, which usually consists of brief generalist advice [29–31].

While health professionals seem to support international guidelines for ending childhood dental caries, they face significant challenges in adequately integrating them into their daily practice. The gap between the theoretical importance of dental prevention and the lack of its clinical implementation requires further investigation. What factors influence carious prevention in children according to health professionals who participate in children’s oral health follow-up? A global overview of the challenges and enablers encountered by clinicians is required to provide relevant information that will help decision-makers or health care teams design and implement oral health preventive actions. To answer this question, we conducted a systematic review that aimed to identify and classify factors perceived by health professionals to be barriers or facilitators to caries prevention in children.

## Method

This systematic review was reported according to the Preferred Reporting Items for Systematic Reviews and Meta-Analyses (PRISMA) guidelines (See Additional file 1) [32]. The study protocol was preregistered on PROSPERO, an international prospective register for systematic reviews (ID: CRD42022304545).

### Searches

The search strategy was designed in collaboration with a medical librarian. Searches were conducted using two major biomedical databases (PubMed and Web of Sciences), as well as a francophone database targeting publications in the humanities and social sciences (Cairn). MeSH terms were used on Medline, and free text terms were used on Web of Science and Cairn (see Additional file 2). The search was conducted with no initial time restriction to March 2021. Since all of the authors are native French speakers, francophone literature that has gone through a complete editing procedure has also been reviewed in addition to articles written in English. No search of grey literature was undertaken. The collection was completed with hand searches of the reference lists of all selected studies.

### Study inclusion and exclusion criteria

After duplicates were removed, two researchers (GL and EM) independently screened the titles and abstracts. Articles not considered relevant to the topic were eliminated, and studies that met the inclusion criteria were collected in full text (consensus of the 2 researchers). In case of disagreement, a third reviewer (MC) was consulted for arbitration. To be included, studies had to focus on barriers and/or facilitators to caries prevention in children and include health professionals as study participants. In this work, barriers and facilitators were defined as factors that help or hinder the implementation of caries prevention with children by health professionals. Qualitative, quantitative, or mixed methods could be included. Conversely, because they were deemed irrelevant to identify barriers and facilitators to caries prevention, guidelines, editorials, and protocols were excluded. To ensure that articles do not appear more than once in the analysis, literature reviews, meta-analyses, and systematic reviews were also eliminated.

In the clinical setting, oral health prevention does not refer to one behavior but to a set of behaviors that health professionals can implement in their clinical practice. It includes screening, risk assessment, counseling, fluoride varnish application, pits and fissure sealants and dental referral. In this regard, caries prevention consists of a comprehensive approach for children. It has been considered that specific prevention measures (fluoride varnish application, fissure sealant, etc.) could not be regarded as a comprehensive prevention strategy and, as such, do not match to the approach the authors wished to take on this issue. Also, they were concerned to include very specific factors that may conflict with those selected as part of a comprehensive approach to prevention. For these reasons, the research team excluded specific studies that covered only one aspect of oral health prevention.

### Assessment of the reporting quality of methodology

The assessment of the reporting quality of the studies’ methodology was conducted independently by two reviewers (GL and EM) using two validated checklists. For qualitative studies, the authors used the Consolidated Criteria for Reporting Qualitative Research (COREQ) [33], a 32-item checklist organized into 3 domains. For quantitative studies, quality assessment was appraised with the strengthening the reporting of observational studies in epidemiology (STROBE) reporting guidelines for cross-sectional studies [34] using a 22-item checklist. The qualitative analysis did not influence study inclusion, but it provided a critical framework for the articles reviewed.

### Data extraction strategy

Data were extracted using the same method as for the study selection. Full texts were analyzed independently by two researchers (GL and EM) with arbitration by a third team member (MC) in case of disagreement. The data collection template included the year of publication, country of the study, primary and secondary objectives of the study, study design, sample size, profession of respondents and main factors identified as barriers or facilitators to caries prevention. For quantitative studies, a factor was considered a barrier when at least 10% of participants reported it as such. This threshold value was decided by consensus of the research team members who considered 10% to be a population-wide significant portion. For qualitative studies, data were collected from participant quotations. A factor was included when both investigators agreed that it was explicitly and unambiguously defined in the text.

### Data synthesis and presentation

Perceived barriers or enablers were classified according to a three-stage process. First, data were sorted based on the 14 domains of the theoretical domains framework (TDF) [35]. The TDF is a comprehensive framework that synthesizes 33 psychological theories related to behavior change and is designed to understand implementation problems. The TDF can be used to conduct various types of studies, including qualitative research, questionnaire studies, evaluations of randomized trials or systematic reviews. To ensure the proper use of the TDF in this systematic review, the authors decided to work according to Atkins and All’s guide [36]. Second, findings were classified depending on whether they were clinician-, patient- or organizational-related. Finally, for each domain, data were reorganized by theme into several subcategories. After data extraction, several meetings with the research team members were organized to synthesize the main factors of the initial analysis into a single framework. This framework was designed using a consensus method.

## Results

### Study selection and characteristics

**Fig. 1:**
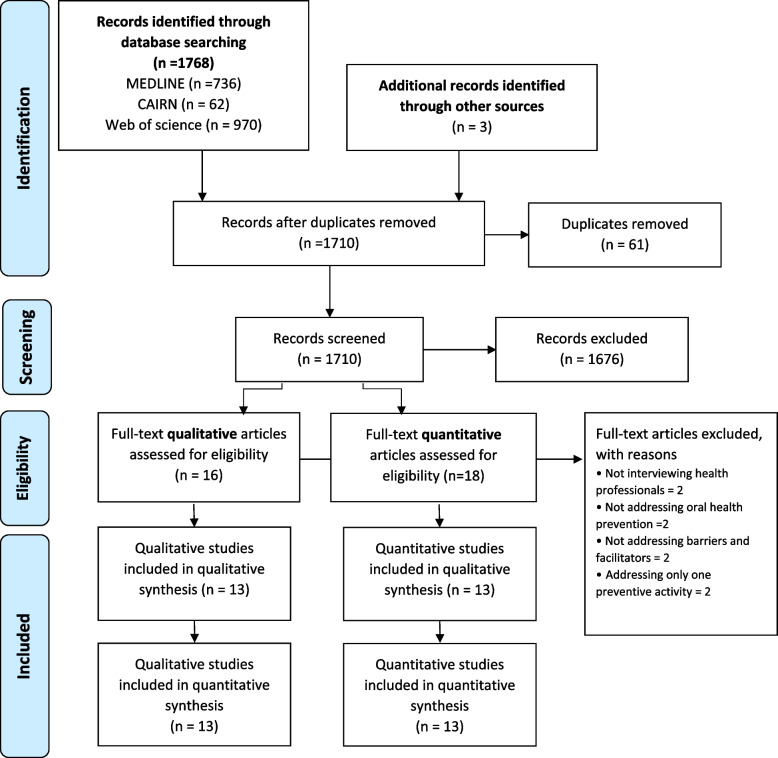
PRISMA flow diagram

The database research found 1768 references (Fig. 1). After duplicates were removed, 1710 studies were screened based on the title and abstract. Of these, 31 were selected for full text examination. After hand searches of the reference lists of all selected studies, 3 additional articles were selected. Ultimately, full text screening was conducted for 34 studies. Eight were excluded (see Additional file 3), and 26 met the inclusion criteria, among which half were qualitative studies [30, 37–48] and half were quantitative studies [24, 25, 49–59]. Methodological quality assessment did not lead to further exclusion of any references.

Among the included papers, 25 were reported in English and one in French [46]. All were published between 2003 and 2019 in nine different countries, including the USA [24, 38, 39, 42, 44, 47, 51, 54, 56, 57], UK [30, 37, 41, 45, 53], Canada [25, 58, 59], Australia [40, 43], Saudi Arabia [49, 50], France [46], Peru [55], Thailand [48] and Taiwan [52] (Table 1). Half of the articles were qualitative studies using individual interviews, focus groups or a combination of both. The other half were cross-sectional studies using a self-report questionnaire. The included studies involved a wide range of health professionals. The most frequently represented professions were dentists (*n* = 12), pediatricians (*n* = 11), nurses (*n* = 9), physicians (*n* = 5), and dental hygienists or dental nurses (*n* = 3).

**Table 1:** Characteristics of included studies

**Author**	**Year**	**Country**	**Study aim**	**Study design**	**Study population**
Al Jameel [49]	2019	Saudi Arabia	1/ To assess the oral health knowledge and practice of pediatricians and pediatric residents in Riyadh 2/ To assess their adherence to American Academy of Pediatrics guidelines for caries-risk assessment and anticipatory guidance for infants and young children 3/ To assess the barriers that affect adherence to these guidelines	Cross-sectional study Self-reported questionnaire	Pediatricians (*n* = 420)
Aljafari [37]	2015	UK	1/ To explore dental practitioners’ experience and views in regard to providing preventive dental care for high caries-risk children 2/ To explore their opinion on what is needed to promote oral health in that cohort	Individual interviews	Dentists (*n* = 18)
Alshunaiber [50]	2019	Saudi Arabia	To assess pediatricians’ and family physicians’ knowledge, attitude and practice towards infants’ oral health and early childhood caries in Riyadh	Cross-sectional study Self-reported questionnaire	Pediatricians, Physicians (*n* = 202)
Bernstein [38]	2016	USA	To identify facilitators and barriers to the integration of oral health into pediatric primary care at health centers to improve problem recognition, delivery of preventive measures, and referral to a dentist	Individual interviews	Physicians, Nurses, Dentists, Administrative staff, Others^a^ (*n* = 39)
Bernstein [39]	2017	USA	1/ To explore the opportunities for interprofessional collaboration (IPC) to improve pediatric oral health in federally qualified health centers 2/ To identify challenges to IPC-led integration of oral health prevention into the well-child visit and to suggest strategies to overcome barriers	Individual interviews	Nurses (*n* = 10)
Cashmore [40]	2011	Australia	1/ To explore the attitudes and beliefs of dental staff about the factors that helped or hindered the establishment and implementation of a hospital-based parent counselling program to manage existing and prevent new carious lesions in children 2/ To explore the influence of the program on the hospital’s reorientation to prevention	Focus groups	Dentists (*n* = 10)
Close	2015	USA	To describe the obstacles encountered by medical providers in North Carolina when incorporating preventive dental services into their practices as part of the Into the Mouths of Babies program	Cross-sectional study Self-reported questionnaire	Pediatricians, Physicians, Nurses (*n* = 231)
Coll	2016	UK	To explore the views of health visitors and school nurses with regard to their role in oral health promotion and their understanding of the issues surrounding the delivery of effective oral health promotion in their daily practice	Focus group	Nurses, Health visitors (*n* = 9)
Dima	2018	Taiwan	1/ To analyze the early childhood caries-related knowledge, attitude and practice of dentists and pediatricians 2/ To identify the pathways through which the knowledge and practice of medical and dental professionals in Taiwan affect their attitude toward medical office-based caries prevention	Cross-sectional study Self-reported questionnaire	Dentists, Pediatric dentists, Pediatricians (*n* = 301)
Elouafkaoui	2014	UK	1/ To determine if further intervention is required to translate the Scottish Dental Clinical Effectiveness Program guidance recommendations into practice 2/ To identify salient beliefs associated with recommended practice	Cross-sectional study Self-reported questionnaire	Dentists (*n* = 87)
Graham	2003	USA	1/ To describe the structure of the oral health program in a university-affiliated hospital 2/ To evaluate staff’s knowledge and attitudes toward oral health 3/ To propose ways to strengthen the incorporation of oral health prevention for children into clinical medical education	Individual interviews	Administrative staff, Nurses, Pediatricians, Dentists (*n* = 17)
Gussy	2006	Australia	To explore the oral health beliefs and practices of primary health care professionals that may act as barriers to the development of a model of shared care for the oral health of pre-school children	Focus groups	Nurses, Dental nurses, Dentists, Pediatricians, Physicians (*n* = 56)
Horowitz	2017	USA	To gain an in-depth understanding of dental hygienists and dentists’ perspectives regarding children’s oral health and what needs to be done to prevent early childhood caries	Focus groups Individual interviews	Dentists, Pediatric dentists, Dental hygienists (*n* = 37)
Lewis	2004	USA	To characterize Washington State pediatricians’ oral health-related educational needs and anticipatory guidance practices	Cross-sectional study Self-reported questionnaire	Pediatricians (*n* = 271)
Lewis	2009	USA	1/ To examine the extent of pediatricians’ current oral health risk assessment and counselling, their perceived ability to perform these tasks, and their attitudes toward their role in oral health risk assessment and counseling 2/ To examine barriers to providing oral health care, including obstacles to young patients obtaining care from a dentist and the influence of the receipt of oral health instruction	Cross-sectional study Self-reported questionnaire	Pediatricians (*n* = 698)
Lewney	2018	UK	To explore how health visitors felt about providing oral health advice and dealing with dental issues during their practice	Individual interviews	Nurses (*n* = 17)
Marquillier	2017	France	To identify the levers and barriers to the development of formalized therapeutic education programs and alternatives	Individual interviews	Dentists, Others^b^ (*n* = 15)
Nelson	2017	USA	To examine how Quality through Technology and Innovation in Pediatrics (QTIP) practices facilitated the adoption of Oral Health Interprofessional Practice into their primary care setting	Individual interviews	Pediatricians, Nurses, Others^c^ (*n* = 22)
Pesaressi	2014	Peru	To identify the barriers that nurses in Lima, Peru, may experience in adopting and implementing a primary oral healthcare program targeted at infants and their caretakers to prevent early childhood caries	Cross-sectional study Self-administered survey	Nurses (*n* = 123)
Prakash	2006	Canada	1/ To assess the knowledge of early childhood caries among pediatricians and family physicians in Canada who provide well care for children younger than three years 2/To examine the proportions of physicians who reported performing oral health-related practices during well care visits for this age group 3/ To determine what oral health education pediatricians and family physicians received during medical and specialty training 4/ To investigate the willingness of these professionals to support oral health promotion activities and barriers to performing these activities	Cross-sectional study Self-reported questionnaire	Pediatricians, Physicians (*n* = 537)
Quinonez	2014	USA	To assess American Academy of Pediatrics fellows’ attitudes and practices related to oral screening, risk assessment, counseling, topical fluoride application, and barriers to dental visits, and examine changes since 2008	Cross-sectional study Self-reported questionnaire	Pediatricians (*n* = 402)
Ruiz	2013	USA	To evaluate the knowledge, comfort, practice behaviors, and perceived barriers of dental hygienists in North Carolina regarding their delivery of oral health preventive services to infants and toddlers	Cross-sectional study Self-reported questionnaire	Dental hygienists (*n* = 758)
Schroth	2013	Canada	1/ To survey dentists about their views on the Free First Visit program 2/ To develop an understanding of their attitudes and practice patterns relating to oral health and first visits among infants and toddlers	Cross-sectional study Self-reported questionnaire	Dentists, Pediatric dentists (*n* = 375)
Stijacic	2009	Canada	To report findings of a mailed survey study about general and pediatric dentists’ practice habits related to oral health in early childhood	Cross sectional study Self-reported questionnaire	Dentists, Pediatric dentists (*n* = 248)
Threlfall	2007	UK	To increase understanding about how and to whom general dental practitioners provide preventive advice to reduce caries in young children	Individual interviews	Dentists (*n* = 93)
Vichayanrat	2013	Thailand	To explore the barriers and facilitating factors among lay health workers (LHWs) and primary care providers (PCPs) in implementing a multi-level program to promote children’s oral health care in a rural Thai community	Individual interviews Focus groups	Lay health workers, Dental nurses, Others^d^ (*n* = 21)

^a^Clinic directors or medical directors or medical assistants

^b^Dental students, pharmacist or teaching manager in patient education or researcher

^c^Practice managers, receptionist, health information technology staff or certified medical assistants

^d^Public health officers or public health technical officers

### Quality of methodology reporting

For the qualitative studies retained, quality assessment using the COREQ checklist showed significant differences in terms of methodological quality (Additional file 4). Overall, the studies provided sufficient detail on aims, the participant selection process, data analysis and reporting. In contrast, more than half of the studies provided poor or no information on the use of a theoretical framework, interviewers’ characteristics, the relationship between the research team and the participants and data saturation. The assessment of quantitative studies showed good methodological quality since most of the items from the STROBE checklist were mentioned for all studies (Additional file 5).

### Findings

A wide range of factors have been identified by health professionals as barriers or facilitators to caries prevention in children (Table 2). The factors were sorted into 3 main categories: clinician-related factors, patient-related factors, and organizational-related factors. For each category, factors were then classified according to the 14 TDF domains.

**Table 2:** Factors perceived by health professionals to be barriers or facilitators to caries prevention in children mapped to the theoretical domains framework (TDF)

TDF domains	Clinician-related factors	Patient-related factors	Organizational-related factors System-related factors
**Knowledge (Awareness of the existence of something)**			
Barriers	• Scientific and procedural knowledge: + Lack of knowledge regarding early childhood caries and child oral health [25, 39, 41, 43, 57–59] + Lack of knowledge regarding preventive activities [46, 51, 52, 55] + Lack of knowledge regarding parents’ education [50] + Lack of knowledge regarding culture-specific oral health information [45] • Lack of familiarity with guidelines *(age at the first dental visit, fluoride recommendations, diet recommendations…)* [38, 39, 44, 48, 49, 56–59] • Misbelief: + Lack of belief in the evidence regarding fluoride efficacy [37] • Knowledge of task environment: + Lack of knowledge regarding dentists’ activity [38, 39] + Lack of awareness of dental services provided locally [45] + Lack of awareness of services available for reducing barriers to dental care (ex: interpreting service) [45] • Illness representations: + Dental caries is not perceived as a chronic disease [46]	• Parents’ scientific knowledge: + Lack of oral health knowledge (*food, hygiene, fluoride, early dental visit)* [25, 37, 39, 43, 44] + Lack of knowledge regarding carious process [25, 44] • Misbelief: + Assumption that parents already had appropriate oral health knowledge [48] • Oral health representations: + Parents do not understand the importance of oral health [37, 47]	
Facilitators	• Scientific knowledge: + Good oral health knowledge [45] • Illness representations: + Non-dental professionals perceive oral health as important [38, 39, 42, 47] + Dental caries is perceived as a major issue that negatively impacts children’s general health and quality of life [43]		
**Skills (Ability or proficiency acquired through practice)**			
Barriers	• Skill development: + Learning how to perform preventive activities is difficult for physicians [52] • Professional skills: + Difficulties applying FV [51] + Lack of counselling skills [48] • Competence: + Dentists believe that hygienists are better at delivering preventive advice [30]	• Poor parental skills: + Poor parenting skills and style (*lack of discipline, negligence…)* [37, 39, 43, 44, 48] + Parents’ low oral health literacy [38, 43, 44] + Parents’ low health literacy [41–43, 47] • High parents’ skills: + If parenting skills are considered sufficient, then prevention activities are not performed [30] • Parents’ inability: + Inability to implement recommendations [30, 37, 54]	
Facilitators	• Importance of empathy in building rapport with parents [40] • Prevention activities are not difficult [53]		
**Social/professional role and identity (A coherent set of behaviors and displayed personal qualities of an individual in a social or work setting)**			
Barriers	• Professional role regarding oral health promotion: + Roles are unclear regarding oral health promotion [42] + Non dental professionals believe that oral health preventive activities are dentists’ responsibility [25, 47, 50] + Non-dental professionals do not think that oral health promotion is their role [25, 43, 45, 55] + Physicians think that some oral health prevention activities are not their role *(identity plaque, tooth brushing education, fluoride varnish application, assess parents’ oral health, parents’ education)* [24, 50, 56] + Dental professionals think that early anticipatory guidance should come from non-dental professionals who have more contact with young children [43] + Primary care providers believe that providing preventive oral health services is lay health workers’ responsibility more than theirs [48] • Professional role regarding children’s care: + Dentists do not want to see young children [43, 57] • Professional boundaries: + Going beyond pediatric clinicians’ field of expertise could have negative consequences for the patient [38]	• Parental disempowerment: + Parents do not take enough responsibility for their children’s oral health care [41, 44]	• Professional role: + The introduction of oral health prevention programs has eroded nurses’ responsibility for providing oral health promotion [41]
Facilitators	• Professional boundaries: + Pediatricians do not think that they would be trespassing on dentists’ job [49] + Hygienists have a closer relationship with the patient than the dentist and take the lead role in patient education [44] • Professional role: + Physicians and family physicians think they play an important role in caries prevention [50, 59] + Physicians think that some oral health prevention activities are their role *(screening, diet education)* [24, 56] + Dentists see themselves in the role of health educators when considering prevention [30] + Nurses accept dental prevention as their responsibility [39] + Nurses think pediatricians have a key role in dental prevention [39] + School nurses believe that health visitors and school teachers have a key role in oral health promotion [41] • Group role: + Involve the entire dental staff in patient education [44] • Commitment: + Oral health promotion in a low-income population is a meaningful mission [42]		
**Beliefs about capabilities (Acceptance of the truth, reality, or validity about an ability, talent, or facility that a person can put to constructive use)**			
Barriers	• Professional confidence: + Dental professionals’ lack of confidence to deliver advice to parents [40, 57] + Dental professionals’ lack of confidence to perform preventive care [57–59] + Non-dental professionals’ lack of confidence to deliver advice to parents [24, 30, 41, 43] + Physicians’ lack of confidence to perform some oral health prevention activities *(screening, tooth brushing education, caries risk assessment, apply fluoride varnish…)* [24, 25, 45, 52, 54, 56]	• Perceived behavioral control: + Young and uncooperative children are unable to accept dental care [37, 52, 57–59] • Parents’ confidence: + Poor diet habits can be explained by poor confidence of parents [43]	
Facilitators	• Professional confidence: + Pediatricians and lay health workers are very confident about delivering advice to parents [48, 56] + Pediatricians are very confident about prescribing fluoride complements [54]		
**Beliefs about consequences (Acceptance of the truth, reality, or validity about outcomes of a behavior in a given situation)**			
Barriers	• Preventive activities’ consequences: + Dentists believe that fluoride complements lead to a higher risk of fluorosis [44]		
Facilitators	• Preventive activities’ consequences: + Oral health prevention activities are perceived to be effective for health behaviors and children’s health [30, 39, 40, 45, 46] + Implementation of preventive programs has a positive impact on the way staff consider prevention [40, 46] • Beliefs: + Prevention activities are perceived to be important [38, 43, 53] + Dental professionals perceive early examinations to be important in preventing dental caries [58, 59]		
**Reinforcement (Increasing the probability of a response by arranging a dependent relationship, or contingency, between the response and a given stimulus)**			
Barriers			
Facilitators	• Consequences: + The implementation of the program allows dental assistants to have more responsibility, with the consequences of more confidence, more satisfaction, more meaning [40]	• Consequents: + Caregivers are grateful and interested in the visits [48]	
**Intention (A conscious decision to perform a behavior or resolve to act in a certain way)**			
Barriers	• Motivation regarding training: + Physicians and family physicians are not interested in receiving additional training [50] + Dentists are not interested in receiving additional training [58] • Motivation regarding preventive activities: + Pediatricians are not willing to perform prevention activities [25, 52] + Dental hygienists are not willing to perform prevention activities [57] + Nurses’ lack of intention to give advice [55] • Motivation regarding children’s care: + Dental professionals are not interested in providing dental care to young children [58] • Resistance to change: + Staff and colleagues’ resistance to change [47, 51, 58]	• Intrinsic motivation: + Parents’ lack of motivation [30, 40]	
Facilitators	• Motivation regarding prevention activities: + The more motivated dentists are, the more likely they are to perform prevention activities [53] + Physicians are interested in oral health prevention [49] • Motivation regarding training: + Dental professionals are interested in receiving additional training [59]		
**Goal (Mental representations of outcomes or end states that an individual wants to achieve)**			
Barriers	• Goal priority: + Oral health prevention is not a priority in dental practice compared to other activities [37, 46] + Non-dental professionals do not consider oral health prevention a priority compared to other activities [41, 42, 51, 54] + Dental and non-dental professionals believe that prevention activities are not sufficiently important [52, 57]	• Goal priority: + Parents do not make oral health a priority compared to other activities [37–39, 43, 44] + Parents do not perceive the need for dental care [24, 25, 50, 56] + Parents are more interested in a curative than a preventive approach [46, 51, 59] + Few parents request the prevention program [58]	
Facilitators	• Target setting: + Oral health should be part of routine anticipatory guidance provided for infants [43]		
**Memory, attention and decision processes (The ability to retain information, focus selectively on aspects of the environment and choose between two or more alternatives)**			
Barriers		• Cognitive overload: + There is too much oral health information for parents to understand it properly [45, 47]	
Facilitators			
**Environmental context and resources (Any circumstance of a person’s situation or environment that discourages or encourages the development of skills and abilities, independence, social competence and adaptive behavior)**			
Barriers		• Social and financial resources: + Low income [30, 44, 47] + Inability to pay [24, 43, 56] + Lack of minimal dental coverage [24, 44, 56] • Cultural barriers: + Language [37, 44, 47] + Culture and social factors affecting parents’ understanding of the role of dentists [37, 44] + At-home oral health practices like the “culture of the bottle” [43, 47] • Other barriers: + Lack of transportation [24, 44] + Parents’ young age [44]	• Resources: + Lack of time [24, 25, 30, 37–39, 41, 42, 44, 46–50, 52, 54, 56, 58, 59] + Lack of supplies [37, 46–48, 59] + Lack of educational tools [30, 39–41, 58, 59] + Lack of financial reward or reimbursement for prevention activities [24, 25, 30, 37, 38, 46, 47, 50, 56, 58, 59] + Lack of human resources [46, 48, 58, 59] • Training system: + Lack of oral health training [24, 25, 37–39, 42, 45, 46, 48, 49, 56, 57] + Lack of training regarding parents’ counselling [40] + Lack of continuing education opportunities [46, 57] • Access to dental care: + Difficulty accessing a dentist for young children [24, 38, 41, 43, 47, 48, 50, 51, 54, 56] + Massive waiting lists at the dental hospital [24, 41, 43] + Lack of a referral system to access a dentist [45] • Political and social environment: + Lack of public policies supporting oral health promotion [37, 48] + Insufficient prevention program funding [46] + Advertising of sugary foods in the child’s environment [37, 44] + Cost of dental care may discourage parents from seeing a dentist [41, 43] + Institutional and legislative complexity [46] + Dental hygienists with the lowest average percentage of medicaid patients were more likely to be in the precontemplation stage to provide preventive care [57] • Interprofessional collaboration: + Lack of interprofessional collaboration [41, 44, 46] + Lack of other primary care providers’ involvement [37, 44] + Lack of collaboration between children’s hospitals and primary care settings [42, 45] + Lack of cross-spatiality communication [40] + Lack of a new profession to delegate preventive activities [46] + Software difficulties limiting cross-specialty communication and interprofessional collaboration [38, 39] • Organizational context: + Different locations of pediatric and dental services [39] + Oral health promotion is not well integrated with existing dental services [41] + Patient education programs are not adapted to private practice [46] • Support + Lack of engagement from the hospital [37] + Lack of peer recognition [46] • Team organization: + High turnover rate in staff [47] + Lack of an oral health champion in charge of the leadership for the implementation of preventive activities [39, 42] + Lack of direct promotion of guidelines [37, 58] + Additional paperwork [42, 44] + The channels through which information can be distributed to parents are not diversified enough [44]
Facilitators	• Organizational culture: + Respondents approve of a collaborative approach to oral health care delivery [38, 39]	• Family environment: + Family vulnerability to further dental disease seems to underpin dentists’ attitudes towards working with these families [40]	• Resources: + Use of educational tools [44, 54] + Giving toothbrushes and toothpaste facilitates home visit activities [48] + Financial reimbursement perceived as sufficient [51] • Training system: + Training programs increase professionals’ knowledge and confidence [48] • Access to dental care: + Partnership between clinics and dental schools or outside private practices to increase access for their patients [38] • Political and social environment: + Private organizations constitute a funding opportunity [46] • Interprofessional collaboration: + Collaboration with other health professionals (nurses and hygienists) [30] + Involving lay workers from the caregivers’ social network [45] + Collaborative meetings help to disseminate knowledge about quality improvement recommendations and share best practices [47] + Team-based communication [47] • Organizational context: + Integrate prevention into the normal course of the department [40] + The period when a child is waiting for dental surgery is an opportune time to intervene with a family that could be difficult to reach [40] • Support: + Support of management and all staff [40] + Upper-level administrators’ involvement is seen as critical in setting the tone for clinic priorities and empowering clinical staff [38] • Team organization: + Designating a team leader to promote oral health [38] + Visiting caregivers at home is the best way to reach caregivers [48]
**Social influences (Interpersonal processes that can cause individuals to change their thoughts, feelings, or behavior effects)**			
Barriers		• Social support: + Influence of significant others in the child’s diet (grandparents and child carers) [43]	
Facilitators			
**Emotion (A complex reaction pattern involving experiential, behavioral, and physiological elements by which the individual attempts to deal with a personally significant matter or event)**			
Barriers	• Negative affect: + Frustration relating to lack of parental responsibility or a lack of standardized practice [37, 41] + Dentists’ disillusion and loss of motivation related to preventive advice not being heeded [30]	• Fear: + Poor attendance due to parents’ fear [43]	
Facilitators			

#### Clinician-related factors

Clinician-related factors were widely discussed in the 26 included studies. The most frequently covered belonged to the following TDF domains: knowledge, professional role and identity, belief in capabilities and beliefs about consequences.

##### Knowledge

Clinicians’ lack of knowledge, especially concerning guidelines [38, 39, 48, 49, 56–59], was the most commonly cited barrier to caries prevention. A lack of scientific knowledge covers various topics, such as early childhood caries and children’s oral health [25, 39, 41, 43, 50, 52, 57–59], preventive activities [46, 51, 52, 55], parents’ education [50] and even culture-specific oral health information [45]. Despite this overall lack of knowledge reported in many studies, professionals’ views on oral health and dental caries constitute two levers for oral health promotion. Oral health is perceived as an essential part of general child health [38, 39, 42, 55], and dental caries is perceived as a major issue that negatively impacts children’s health and quality of life [43].

##### Professional role and identity

The professional role and responsibility for caries prevention in children is a major theme in our analysis and is discussed by more than half of the selected articles. Data extraction reveals that roles are confused regarding oral health promotion since it is difficult to clearly understand which health professionals (dental or nondental) are responsible for it. Some respondents express a strong opinion on this matter, stating that oral health promotion is not their role [25, 43, 45, 47, 50, 55]. In several studies, physicians, pediatricians and nurses argue that preventive dental activities are dentists’ responsibility [25, 47, 50]. Conversely, other papers report that some dental professionals do not want to see children and believe that early anticipatory guidance should come from nondental professionals who have more contact with young children [43, 57]. Our analysis also shows more moderate views, with physicians who believe that their role is restricted to specific preventive activities, such as counseling or screening [24, 50, 56]. In addition, some health professionals talk about sharing the responsibility for prevention with other actors in a better position for its implementation than themselves. In these cases, the responsibility is transferred to dentists, lay health workers, health visitors or teachers, for example [41, 48].

##### Belief about capabilities

Lack of confidence about performing some preventive activities or advising parents on their child’s oral health is reported more frequently by nondental professionals [24, 25, 41, 43, 45, 52, 54, 56] than dental professionals [40, 57–59]. However, the data provide conflicting information, with some respondents feeling confident in delivering advice to parents or in prescribing fluoride supplements [48, 54, 56].

##### Belief about consequences

The factors identified in this domain are mainly levers for caries prevention in children. Health professionals’ perception of preventive activities seems to support oral health promotion since they are considered important [38, 43, 53, 58, 59] and lead to positive changes in health behaviors and children’s health [30, 39, 40, 45, 46].

##### Intention

Factors related to this domain are reported less frequently. However, several studies point to a lack of motivation among professionals in providing dental care for children [58], carrying out preventive activities [25, 52, 55, 57], receiving additional training regarding oral health and caries prevention [50, 58] or more generally changing clinical practices [47, 51, 58].

##### Goal

In some papers, respondents question the importance of oral health prevention. In these articles, dental and nondental professionals state that oral health prevention is not considered a priority compared to other activities [37, 41, 42, 46, 51, 54, 57].

#### Patient-related factors

Eleven TDF domains report patient-related factors. The most frequently reported domains are knowledge, skills, goal and environmental context and resources.

##### Knowledge and skills

In several studies, clinicians suggest that parents’ lack of scientific knowledge on oral health and carious process is an important barrier to maintaining good oral health in children [25, 37, 39, 43, 44]. For some of them, parents’ poor knowledge would explain their inability to understand the importance of good oral health [44, 47]. In several studies, parental skills are also perceived as a barrier to caries prevention. Health professionals explain inappropriate oral health habits based on parents’ lack of authority and reluctance to be firm with their children [37, 39, 43, 44, 48] as well as their poor health and oral health literacy [38, 41–44, 47]. Parents are also considered unable to implement healthy behavior since they do not adhere to the recommendations made [30, 37, 56].

##### Goal

Goal priority is described by health professionals as an important issue for the development of preventive activities. Clinicians believe that parents do not make oral health a priority compared to other activities [37–39, 43, 44]. More precisely, they think that parents do not perceive the need for dental care [25, 50, 56] and the necessity of a preventive approach to dental caries management [46, 51, 58, 59].

##### Environmental context and resources

Low-income families are seen as facing the most challenges [30, 44, 47]. Regardless of the characteristics of the health system, these families are more likely to forego dental care because of their inability to pay due to low resources and the absence of minimal dental coverage [24, 43, 44, 56]. In several qualitative studies, cultural factors are described as another element that complicates oral health promotion. The sociocultural background of these families would partly explain poor oral health practices at home as well as parents’ understanding of dentists’ roles and the importance of good oral health [37, 43, 44, 47]. Finally, language barriers are also seen as an important factor that affects the quality of communication and care [37, 44, 47].

#### Organizational-related factors

All organizational-related factors considered in the analyses fall within the “Environmental Context and Resources” TDF domain.

##### Environmental context and resources

Of all the TDF categories, this domain includes the largest number of factors that cover a wide range of topics. Different types of resources are mentioned as key obstacles in caries prevention. A lack of time in clinical practice is the most frequently reported factor in this review [24, 25, 30, 37–39, 41, 42, 44, 46–50, 52, 54, 56, 58, 59]. Financial resources are also frequently debated, with respondents criticizing the lack of financial reward or reimbursement for preventive activities [24, 25, 30, 37–39, 46, 47, 56, 58, 59]. Difficulties in implementing prevention activities are also explained by poor material resources [30, 37, 39–41, 46–48, 58, 59] due to a lack of supplies (e.g., fluoride varnish, tooth brushes) or educational tools as well as insufficient human resources due to staff shortages [46, 48, 58, 59]. The education system’s weaknesses also appear as a recurrent theme in the analysis. Health professionals complain about an overall lack of oral health training [24, 25, 37–39, 42, 46, 48, 49, 56, 57], poor training in counseling techniques [40] and insufficient continuing education opportunities [46, 57]. Difficulty in accessing dental care is another major barrier cited in more than half of the included studies. More specifically, non-dental professionals report great difficulties in referring young children to dentists[24, 38, 41, 43, 47, 48, 50, 51, 54, 56] and massive waiting lists to access dental hospitals [24, 41, 43]. The lack of a referral system would contribute to poor access to dental care and prevention [46, 57]. The development of a partnership between clinics and dental schools or outside private practices would make this easier [38].

Further environmental and organizational factors can be added to the barriers previously described. An unfavorable political environment would hinder oral health prevention development because of a lack of support from public policies [37, 48], legislative complexity [46] and insufficient funding [46]. Private organizations are presented in one study as a sensible funding option [46]. Concerning the health care organization, prevention programs are described as inappropriate for the way in which dental services and private practice operate [41, 46]. Paradoxically, integrating prevention while maintaining the normal course of the service is presented as a key element to successfully implementing new programs [40]. Lack of support from the hierarchy and from peers is also seen as problematic in several articles [37, 46]. This is remarkable because some respondents state that the support of management and all staff as well as the involvement of upper-level administrators is critical in setting the tone for clinic priorities and empowering clinical staff [38, 40]. At the health care team level, interprofessional collaboration and communication are often criticized and considered insufficient between the different actors in charge of children’s oral health [37, 40–42, 44–46]. According to some clinicians, the organization of the health care teams is not correctly optimized since they are not headed by an “oral health champion” in charge of the leadership for the implementation of preventive activities [39, 42]. Health professionals state that leadership is crucial for the implementation and sustainability of prevention programs.

### Data synthesis

**Fig. 2:**
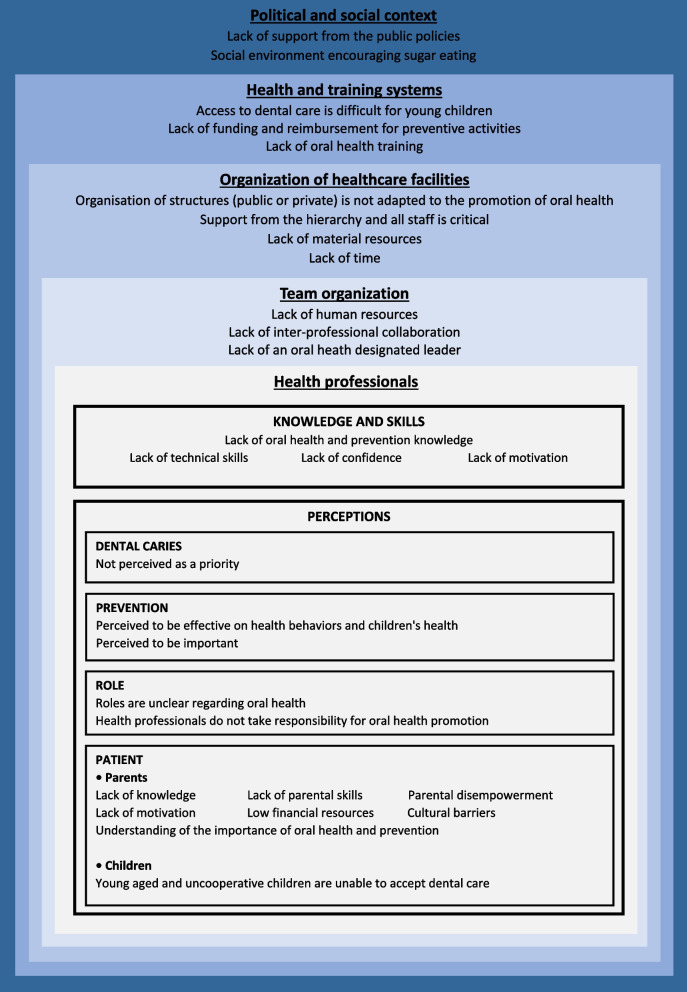
Data synthesis

The main factors from the TDF analysis were extracted and classified into 5 categories: “Political and social context”, “Training and health systems”, “Organization of health care facilities”, “Team organization” and “Health professionals”. Figure 2 highlights the fact that barriers to and facilitators of caries prevention in children involve all stages of the health care system, from public policies to health professionals’ opinions.

## Discussion

### Main results

In this systematic review of 26 studies, health professionals reported many challenges to caries prevention in children. The barriers identified in this systematic review are varied and systemic and involve all stages of the health care system: the political and social context, health system organization, health care facilities organization, health care team organization and health professionals’ skills and opinions. Health professionals frequently point to organizational barriers, particularly lack of time, poor material resources, inadequate funding or reimbursement, insufficient oral health training and difficulty accessing dental care. Parents would constitute another obstacle to children’s oral prevention. Due to their lack of knowledge, parenting skills, and health literacy, they may not recognize their child’s oral health as a priority. Health professionals are also questioned because of their lack of dental knowledge, lack of self-confidence, and unclear understanding of their role in promoting oral health.

### Comparison to the literature

To our knowledge, this is the first systematic review to investigate health professionals’ perspectives on the barriers to and facilitators of caries prevention in children. In 2017, a scoping review on a related topic was conducted by Harnagea et al. [60] to identify the factors that influence the integration of oral health into primary care. The main barriers identified in their study were very similar to those found in our research: a lack of political leadership and health care policies, lack of time, lack of staff, limited knowledge and competencies and insufficient oral health education. By applying a multilevel analysis theoretical framework [61], the authors also demonstrated, as we did in our study, that the various factors mentioned by health professionals involved all stages of the health care system (macro, meso and micro levels).

Previous quality improvement projects have been conducted to increase the delivery of oral health care and prevention within clinical practice. By improving payment for preventive dental services, some of these projects have sought to address one of the most widely cited barriers in our review [62–65]. These studies show a positive but limited impact of funding measures on the provision of preventive dental care. Although more generous payment policies are needed, they are not sufficient to ensure the widespread implementation of preventive services at the organizational and practical levels. Other initiatives focus on improving health professional oral health education, which was also identified as a major barrier in our research [66, 67]. These studies report a moderate effect of oral health training on the provision of preventive dental services. Some authors [24, 51] even state that there is no connection between prior training or knowledge in the field of oral health and the delivery of preventive care. Health professionals’ training is also a necessary but insufficient factor for clinical changes. These findings indicate the importance of actions addressing multiple barriers. Several interventions [68, 69] to address various types of barriers have resulted in a significant improvement in the delivery of preventive dental services (fluoride varnish (FV) application and dental referral). In a few months, the FV application rate rose by more than 75% in facilities where the programs had been implemented. In addition to increasing reimbursement and professional training, these projects included hiring a project manager, developing education brochures and posters, providing an updated list of local dentists, and involving care assistants to share the workflow.

### Areas for future research

The organizational barriers identified in our study are not specific to oral health prevention. The same difficulties in prevention implementation are discussed in other systematic reviews focusing on different noncommunicable diseases (obesity [70], diabetes [71], mental illness [72, 73], cardiometabolic diseases [74], and asthma [75]).

Regardless of the disease, health professionals report struggling with time and workload, insufficient funding, lack of staff, shortage of materials, poor collaboration with specialists, inadequate training, confusion about roles and responsibilities, and a lack of leadership and management. The complexity of integrating prevention into clinical practice is not specific to dental caries and appears to apply equally to the prevention of a wide range of noncommunicable diseases. This topic could be further investigated through another systematic review studying barriers and facilitators shared by different noncommunicable diseases. This research provides a comprehensive view of the difficulties encountered by health professionals and encourages policy-makers to reconsider the health care system’s organization to better integrate prevention into patients’ care pathway.

Among all of the factors discussed in this systematic review, health professionals commonly mention parents as a barrier to effective oral health prevention for children. Parents of children with dental caries are described as lacking oral health knowledge, parental skills, motivation, and authority. Obesity research has shown that health professionals’ negative perceptions of their patients could affect disease management quality due to shorter consultations, less respectful communication, and a less patient-centered approach [76]. Therefore, it would be useful to undertake additional research to identify health professionals’ perceptions of children with dental caries and their families and how these perceptions could influence children’s quality of care.

Our study shows that the implementation of individual caries prevention in the medical setting is a global issue involving numerous, varied, and systemic barriers. Improving individual prevention will likely require a wide range of interventions addressing different types of factors. Therefore, the development of caries prevention in health care settings is likely to follow a lengthy and challenging implementation process. In this context, it appears critical that researchers and policy-makers continue to work on diversified prevention strategies, such as collective measures. While fluoride has long been used at an international level [77], other strategies are still underexploited and warrant further investigation. This is the case with several sugar-lowering measures recommended by the World Health Organization [2]: 1) taxation of sugar-sweetened beverages and foods with high free sugar content; 2) clear nutrition labeling about sugars contained in a product; and 3) regulation of marketing and advertising of food and beverages high in free sugars to children.

### Strengths and limitations

The use of the theoretical domain framework (TDF) in the development of the data extraction and analysis template is one of our study’s strengths. Many different behavioral change theories exist, and others could have been used as theoretical frameworks for this study. These psychological theories involve a wide range of constructs, and their complexity can sometimes make them difficult to apply in a research setting. In this context, selecting and applying a theoretical framework may be challenging for researchers. The TDF offers a reasonable answer to these challenges by providing a comprehensive and practical framework that synthesizes 33 psychological theories and 128 constructs. Developed in 2005 by Michi et al. [78], the TDF was modified and validated to strengthen its structure and content [35]. This model is now commonly used by researchers to assess health-related behavior and implementation problems [79]. In this review, factors were categorized following the 14 TDF domains and then sorted according to whether they were clinician-, patient-, or organization-related. This two-step method provided a clear understanding of the factors that affect oral health prevention in children. Additionally, the relevance of our approach is reinforced by its use in other recent systematic reviews studying implementation difficulties in the medical setting [71, 80, 81]. Another strength of our work is that it included studies that questioned all primary care professionals engaged in children’s dental health follow-up. Moreover, our analysis provides a well-distributed number of studies pooling dental professionals and others pooling nondental professionals. Our findings thus provide an overall view of the challenges that limit the implementation of pediatric caries prevention.

Regarding limitations, the search strategy was limited to 3 international databases with no search of the grey literature, and only articles written in English and French were considered for the analysis. The databases were chosen after discussions with a medical librarian. Prior to the investigation, more databases have been explored (especially Scopus). Because the findings from Scopus contained an extensive number of duplicates and articles irrelevant to the research issue, the study was eventually restricted to three databases (PubMed, Web of Sciences, and Cairn). The exclusion of grey literature may also be regarded as an important constraint, since the inclusion of unpublished data can reduce the effect of publication biais. However, the variability of this literature’s editorial process does not always ensure reliable data. Moreover, it has been found that unpublished studies rarely influence the results and conclusions of a review [82]. For these reasons, the research team and the professional librarian involved in the project agreed that the use of three databases, in combination with a rigorous manual search, would be sufficient to guarantee the quality of the research. Although a few references may have been overlooked, it is likely that the review included the most relevant references. Additionally, investigators did not use specific tools for the evaluation of the methodological quality of studies (such as the Critical Appraisal Skills Program criteria Checklists). However, a reporting quality assessment was consciously and independently conducted by the two main reviewers (GL and EM) using the COREQ and STROBE checklists. Two tables detailing the completion of these checklists are supplied, providing a good overview of each article’s reporting quality strengths and weaknesses (Supplementary material). In addition, the research team members decided that in quantitative studies, a factor was considered a barrier when at least 10% of participants reported it as such. This arbitrary choice of a 10% cutoff number may be considered a methodological limitation. Given its greater significance at a population level, a 20% threshold value may have seemed more reasonable. This issue was discussed extensively during the study design. The researchers finally decided to record, during the data extraction process, all the factors that 10 to 20% of the participants considered barriers. A small number of these factors were identified during the data analysis. The research team consequently chose to maintain a 10% cutoff since a 20% value would not change the results. Moreover, there is no consensus in the literature concerning this cutoff value. Finally, some critical components may be underrepresented in our findings. The methods used in the studies included in our analysis could have influenced the participants’ responses and led them to emphasize some factors more than others. The use of closed-ended questions in quantitative studies means that the factors discussed are suggested to the participants by the investigators. In these studies, participants are not given the opportunity to cite factors that are not mentioned in the questionnaires. As a result, some factors are heavily cited in our review, which may lead to the mistaken assumption that some factors are more important than others when they are simply overrepresented in quantitative studies. However, our study included a significant amount of qualitative research, which resulted in a wide range of factors being discussed. To strengthen the results of this systematic review and address this bias, future research may conduct a quantitative study using the TDF questionnaire [83, 84]. If tailored to the context of pediatric caries prevention, this questionnaire could be used to independently assess the influence of the 14 TDF domains on clinicians’ behaviors. This study could help researchers identify the most relevant levers for designing evidence-based interventions to improve health professionals’ integration of caries prevention in clinical practice.

## Conclusion

This systematic review identified a diverse set of barriers and facilitators to caries prevention in children across nearly all TDF domains. Although organizational factors were the most frequently reported in our analysis, individual factors (clinician- or patient-related) were also mentioned as playing an important role. This study emphasized the systemic character of the oral prevention challenge. This research aimed to provide a comprehensive view of the difficulties encountered by health professionals and to encourage policy-makers to reconsider the organization of the health care system to better integrate prevention into patients’ care pathway.

### Supplementary Information

**Additional file 1.** PRISMA 2020 Checklist.

**Additional file 2.** Search strategies on Medline, Web of Science and Cairn.

**Additional file 3.** Excluded full-text articles and references.

**Additional file 4.** Quality of methodology reporting of qualitative studies included in the analysis.

**Additional file 5.** Quality of methodology reporting of quantitative studies included in the analysis.

## Data Availability

The datasets used and/or analyzed during the current study are available from the corresponding author on reasonable request.

## Abbreviations

TDF: Theoretical domain framework
FV: Fluoride varnish
COREQ: Consolidated criteria for reporting qualitative research
STROBE: Strengthening the reporting of observational studies in epidemiology
