# Supplementary material for: Factors perceived by health professionals to be barriers or facilitators to caries prevention in children: a systematic review
Source: BMC Oral Health. 2023 Oct 19;23:767. doi: 10.1186/s12903-023-03458-1 (PMC10585780; doi:10.1186/s12903-023-03458-1)
Supplement: Supplementary file 2 — Additional file 2. Search strategies on Medline, Web of Science and Cairn. [file 12903_2023_3458_MOESM2_ESM.docx]

**Additional File 2 - Search strategies on Medline, Web of Science and Cairn**

| **Medline** |
| --- |
| (((dental caries[MeSH Terms]) OR (oral health[MeSH Terms])) AND ((child[MeSH Terms]) OR (infant[MeSH Terms]))) AND ((attitude of personnel [MeSH Terms]) OR (health knowledge, attitudes, practice[MeSH Terms])) |
| **Web of Sciences** |
| (Dental Caries* OR oral health) AND (infant*OR child* OR pediatr*) AND (preven* OR patient educa*) AND ( dentis*  OR  physician*  OR  pediatr*  OR  nurs* ) |
| **Cairn** |
| Carie* dentaire* ET prévention* ET enfant* |
