# Supplementary material for: Factors perceived by health professionals to be barriers or facilitators to caries prevention in children: a systematic review
Source: BMC Oral Health. 2023 Oct 19;23:767. doi: 10.1186/s12903-023-03458-1 (PMC10585780; doi:10.1186/s12903-023-03458-1)
Supplement: Supplementary file 3 — Additional file 3. Excluded full-text articles and references. [file 12903_2023_3458_MOESM3_ESM.docx]

**Additional file 3. Excluded full-text articles and references**

**Excluded full-text articles and reasons for exclusion (n=8)**

| First author | Year of publication | Reason for exclusion |
| --- | --- | --- |
| **AlYoussef (1)** | 2013 | Not addressing barriers and facilitators |
| **Arpalathi (2)** | 2012 | Not addressing barriers and facilitators |
| **Dahlberg (3)** | 2014 | Addressing only one specific activity |
| **Karasz (4)** | 2005 | Not addressing oral health prevention |
| **Lewis (5)** | 2005 | Addressing only one specific activity |
| **Masoe (6)** | 2015 | Not interviewing health professionals |
| **Pine (7)** | 2004 | Not addressing oral health prevention |
| **Sams (8)** | 2013 | Not interviewing health professionals |

**References of excluded full-text articles**

1. AlYousef Y, Damiano P, Weber-Gasparoni K, Qian F, Murph J, Nothwehr F. Medical students’ child oral-health-related knowledge, practices and attitudes. Eur J Dent Educ Off J Assoc Dent Educ Eur. 2013;17(4):218‑24.

2. Arpalahti I, Järvinen M, Suni J, Pienihäkkinen K. Acceptance of oral health promotion programmes by dental hygienists and dental nurses in public dental service. Int J Dent Hyg. 2012;10(1):46‑53.

3. Dahlberg D, Hiott DB, Wilson CC. Implementing Pediatric Fluoride Varnish Application in a Rural Primary Care Medical Office: A Feasibility Study. J Pediatr Health CARE. 2019;33(6):702‑10.

4. Karasz A, Patel V, Ranasinghe S, Chaudhuri K, McKee D. Preventing caries in young children of immigrant Bangladeshi families in New York: perspectives of mothers and paediatricians. Community Dent Health. 2014;31(2):80‑4.

5. Lewis C, Lynch H, Richardson L. Fluoride varnish use in primary care: what do providers think? Pediatrics. 2005;115(1):e69-76.

6. Masoe AV, Blinkhorn AS, Taylor J, Blinkhorn FA. Assessment of the management factors that influence the development of preventive care in the New South Wales public dental service. J Healthc Leadersh. 2015;7:1‑11.

7. Pine CM, Adair PM, Burnside G, Nicoll AD, Gillett A, Borges-Yáñez SA, et al. Barriers to the treatment of childhood caries perceived by dentists working in different countries. Community Dent Health. 2004;21(1 Suppl):112‑20.

8. Sams LD, Rozier RG, Wilder RS, Quinonez RB. Adoption and Implementation of Policies to Support Preventive Dentistry Initiatives for Physicians: A National Survey of Medicaid Programs. Am J PUBLIC Health. 2013;103(8):E83‑90.
