# Supplementary material for: Factors perceived by health professionals to be barriers or facilitators to caries prevention in children: a systematic review
Source: BMC Oral Health. 2023 Oct 19;23:767. doi: 10.1186/s12903-023-03458-1 (PMC10585780; doi:10.1186/s12903-023-03458-1)
Supplement: Supplementary file 4 — Additional file 4. Quality of methodology reporting of qualitative studies included in the analysis. [file 12903_2023_3458_MOESM4_ESM.docx]

**Additional File 4: Quality of methodology reporting of qualitative studies included in the analysis**

|  | **Reporting criteria** | **Aljafari 2015** | **Bernstein 2016** | **Bernstein 2017** | **Cashmore 2011** | **Coll 2016** | **Graham 2003** | **Gussy 2006** | **Horowitz 2017** | **Lewney 2018** | **Marquiller 2017** | **Nelson 2017** | **Threlfall 2007** | **Vichayanrat 2013** |
| --- | --- | --- | --- | --- | --- | --- | --- | --- | --- | --- | --- | --- | --- | --- |
| **Personal characteristics** | 1 Interviewer/facilitator |  |  |  |  |  |  |  |  |  |  |  |  |  |
|  | 2 Credentials (PhD,MD,E.g.) |  |  |  |  |  |  |  |  |  |  |  |  |  |
|  | 3 Occupation |  |  |  |  |  |  |  |  |  |  |  |  |  |
|  | 4 Gender |  |  |  |  |  |  |  |  |  |  |  |  |  |
|  | 5 Experience and training |  |  |  |  |  |  |  |  |  |  |  |  |  |
| **Relationship with participants** | 6 Relationship established |  |  |  |  |  |  |  |  |  |  |  |  |  |
|  | 7 Participant knowledge of the interviewer |  |  |  |  |  |  |  |  |  |  |  |  |  |
|  | 8 Interviewer characteristics |  |  |  |  |  |  |  |  |  |  |  |  |  |
| **Theoretical framework** | 9 Methodological orientation and theory |  |  |  |  |  |  |  |  |  |  |  |  |  |
| **Participant selection** | 10 Sampling |  |  |  |  |  |  |  |  |  |  |  |  |  |
|  | 11 Method of approach |  |  |  |  |  |  |  |  |  |  |  |  |  |
|  | 12 Sampling size |  |  |  |  |  |  |  |  |  |  |  |  |  |
|  | 13 Non-participation |  |  |  |  |  |  |  |  |  |  |  |  |  |
| **Setting** | 14 Setting of data collection |  |  |  |  |  |  |  |  |  |  |  |  |  |
|  | 15 Presence of non-participants |  |  |  |  |  |  |  |  |  |  |  |  |  |
|  | 16 Description of sample |  |  |  |  |  |  |  |  |  |  |  |  |  |
| **Data collection** | 17 Interview guide |  |  |  |  |  |  |  |  |  |  |  |  |  |
|  | 18 Repeat interview |  |  |  |  |  |  |  |  |  |  |  |  |  |
|  | 19 Audio/visual recording |  |  |  |  |  |  |  |  |  |  |  |  |  |
|  | 20 Field notes |  |  |  |  |  |  |  |  |  |  |  |  |  |
|  | 21 Duration |  |  |  |  |  |  |  |  |  |  |  |  |  |
|  | 22 Data saturation |  |  |  |  |  |  |  |  |  |  |  |  |  |
|  | 23 Transcripts returned |  |  |  |  |  |  |  |  |  |  |  |  |  |
| **Data analysis** | 24 Number of data coders |  |  |  |  |  |  |  |  |  |  |  |  |  |
|  | 25 Description of the coding tree |  |  |  |  |  |  |  |  |  |  |  |  |  |
|  | 26 Derivation of themes |  |  |  |  |  |  |  |  |  |  |  |  |  |
|  | 27 Software |  |  |  |  |  |  |  |  |  |  |  |  |  |
|  | 28 Participant checking |  |  |  |  |  |  |  |  |  |  |  |  |  |
| **Reporting** | 29 Quotations presented |  |  |  |  |  |  |  |  |  |  |  |  |  |
|  | 30 Data and findings consistent |  |  |  |  |  |  |  |  |  |  |  |  |  |
|  | 31 Clarity of major themes |  |  |  |  |  |  |  |  |  |  |  |  |  |
|  | 32 Clarity of minor themes |  |  |  |  |  |  |  |  |  |  |  |  |  |

|  | Component was used |
| --- | --- |
|  | Component was not used |
|  | The information provided was incomplete |
