# Supplementary material for: Factors perceived by health professionals to be barriers or facilitators to caries prevention in children: a systematic review
Source: BMC Oral Health. 2023 Oct 19;23:767. doi: 10.1186/s12903-023-03458-1 (PMC10585780; doi:10.1186/s12903-023-03458-1)
Supplement: Supplementary file 5 — Additional file 5. Quality of methodology reporting of quantitative studies included in the analysis. [file 12903_2023_3458_MOESM5_ESM.docx]

**Additional File 5: Quality of methodology reportingof quantitative studies included in the analysis**

|  | **Reporting criteria** | **Al Jameel 2019** | **Alshunaiber 2018** | **Close 2015** | **Dima 2018** | **Elouafkaoui 2014** | **Lewis 2004** | **Lewis 2009** | **Pesaressi 2014** | **Prakash 2006** | **Quinonez 2014** | **Ruiz 2013** | **Schroth 2013** | **Stijacic 2009** |
| --- | --- | --- | --- | --- | --- | --- | --- | --- | --- | --- | --- | --- | --- | --- |
| **Titre and abstract** | 1a Study design |  |  |  |  |  |  |  |  |  |  |  |  |  |
|  | 1b Summary |  |  |  |  |  |  |  |  |  |  |  |  |  |
| **Introduction** | 2 Background/rationale |  |  |  |  |  |  |  |  |  |  |  |  |  |
|  | 3 Objectives |  |  |  |  |  |  |  |  |  |  |  |  |  |
| **Methods** | 4 Study design |  |  |  |  |  |  |  |  |  |  |  |  |  |
|  | 5 Setting |  |  |  |  |  |  |  |  |  |  |  |  |  |
|  | 6 Participants |  |  |  |  |  |  |  |  |  |  |  |  |  |
|  | 7 Variables |  |  |  |  |  |  |  |  |  |  |  |  |  |
|  | 8 Data sources/ measurement |  |  |  |  |  |  |  |  |  |  |  |  |  |
|  | 9 Bias |  |  |  |  |  |  |  |  |  |  |  |  |  |
|  | 10 Study size |  |  |  |  |  |  |  |  |  |  |  |  |  |
|  | 11 Quantitative variables |  |  |  |  |  |  |  |  |  |  |  |  |  |
|  | 12 Statistical methods |  |  |  |  |  |  |  |  |  |  |  |  |  |
| **Results** | 13 Participants |  |  |  |  |  |  |  |  |  |  |  |  |  |
|  | 14 Descriptive data |  |  |  |  |  |  |  |  |  |  |  |  |  |
|  | 15 Outcome data |  |  |  |  |  |  |  |  |  |  |  |  |  |
|  | 16 Main results |  |  |  |  |  |  |  |  |  |  |  |  |  |
|  | 17 Other analysis | NA | NA | NA |  | NA | NA | NA | NA |  |  |  |  |  |
| **Discussion** | 18 Key results |  |  |  |  |  |  |  |  |  |  |  |  |  |
|  | 19 Limitations |  |  |  |  |  |  |  |  |  |  |  |  |  |
|  | 20 Interpretation |  |  |  |  |  |  |  |  |  |  |  |  |  |
|  | 21 Generalisability |  |  |  |  |  |  |  |  |  |  |  |  |  |
| **Other information** | 22 Funding |  |  |  |  |  |  |  |  |  |  |  |  |  |

|  | Component was used |
| --- | --- |
|  | Component was not used |
| NA | No further analysis required |
|  | The information provided was incomplete |
